# Feasibility of Functional Electrical Stimulation-Assisted Neurorehabilitation following Stroke in India: A Case Series

**DOI:** 10.1155/2012/830873

**Published:** 2012-08-01

**Authors:** Bhawna Khattar, Alakananda Banerjee, Rajsekhar Reddi, Anirban Dutta

**Affiliations:** ^1^Department of Physiotherapy and Rehabilitation, Max Super Speciality Hospital, Saket, New Delhi, India; ^2^Department of Neurology, Max Super Speciality Hospital, Saket, New Delhi, India; ^3^Department of Clinical Neurophysiology, Georg-August-University, Goettingen, Germany

## Abstract

Functional Electrical Stimulation (FES) facilitates ambulatory function after paralysis by electrically activating the muscles of the lower extremities. The Odstock Dropped Foot Stimulator (ODFS, Odstock, UK) called ODFS Pace, was used for heel-switch triggered FES-assisted walking. The ODFS is recommended as an intervention for neurologically impaired gait in the Royal College of Physicians (UK) Clinical Guidelines on Stroke. Based on the guidelines by the National Institute of Clinical Excellence (NICE, UK), we started first clinical study in India on ODFS Pace as an orthotic intervention for daily use. In this preliminary study, we also investigated improvement in volitional walking following 6 sessions (3 times per week, for 2 weeks) of 30 minutes of FES-assisted treadmill walking on 7 chronic (>6 months after stroke) stroke survivors. We found that short-duration, moderately intensive FES-assisted gait therapy improved volitional gait in 3 out of 7 stroke survivors suffering from foot drop. Even in absence of improvement in volitional walking, there were no adverse effects and the subjects found heel-switch triggered FES-assisted walking mostly “easy” (6 out of 7). Therefore FES is promising as an orthotic intervention for daily use; however, tailoring the intensity and/or frequency based on patient's ability may make it viable as a therapeutic intervention.

## 1. Introduction

The World Health Organization (WHO) defines stroke as “the rapid development of clinical signs and symptoms of a focal neurological disturbance lasting more than 24 hours or leading to death with no apparent cause other than vascular origin” [1]. Worldwide, 15 million people suffer a stroke every year, and stroke is the second leading cause of disability after dementia. India-centered studies have presented a prevalence rate of 27–34/100,000 in the 35–44 age group to 822–1116/100,000 in the 75+ age group [2–4]. The prevalence of stroke in younger individuals is higher in India as compared with high-income nations [5]. The burden of stroke on the quality of life is much greater in this younger age group of stroke survivors [5–7]. Moreover stroke drastically affects the economical productivity for that younger age group adding further to the overall disease burden [5] and therefore early rehabilitation to alleviate disability has a special significance in developing countries.

The disability due to poor ability to walk in stroke patients is frequently caused by the “foot drop” symptom that prevents the patient from being able to raise the foot during the swing phase of walking [8]. The foot slaps down on the ground after heel strike and the toe drags during the swing phase. In addition to that the patients often lack push-off power due to weakness in ankle plantar flexors (like gastrocnemius muscle), which not only reduces walking speed but also reduce ankle dorsiflexion angles during the early swing phase of gait [9]. Goldberg et al. have shown that the ankle plantar flexors are important for knee flexion velocity during push-off [10]. This leads to insufficient foot clearance that puts the patient at risk for stumbling and falling [9]. Consequently impairment of walking has been mentioned most frequently (39–90%) as the most important disabling condition in community-dwelling stroke survivors, and improving walking ability has been found to be the best way to reduce dependency [11, 12]. Almost 30% of patients who survive stroke are affected by foot drop, where the ankle dorsiflexors (e.g., tibialis anterior muscle) are functionally impaired [8, 9]. 

The current rehabilitation techniques following stroke are largely based on some variation of the Bobath concept which relies primarily on inhibiting spasticity and other abnormal responses through physiotherapy [13]. However there is no evidence of superiority of Bobath concept on improvements in sensorimotor control of upper and lower limb, dexterity, mobility, activities of daily living, health-related quality of life, and cost-effectiveness following stroke, and no evidence is available for the superiority of any other current approach [14]. Therefore, there is an urgent need to incorporate additional interventions based on recent findings from animal and human studies on task-specific motor learning and neuroplasticity into the framework of rehabilitation following stroke [15]. The challenge is in providing an orthotic intervention that facilitates community ambulation and in long term promotes normal recovery of gait so that the stroke survivor can wean away from the orthosis.

Functional-electrical-stimulation-(FES-) based orthosis has been shown to enhance walking abilities, increasing gait speed while lowering effort and has only recently developed into a therapeutic intervention for stroke rehabilitation [8, 16–25]. FES involves electrical stimulation of nerves and muscles with continuous short pulses of electrical current [26]. Hausdorff and Ring showed that the number of falls reduced significantly in hemiparetic patients who used FES to correct foot drop [23]. Although FES provides orthotic benefit, the “carry over” effect on volitional movement and the required dosing for long-term therapeutic benefit have not been investigated thoroughly. Daly et al. showed that gait training with FES had significant additive effect on gait coordination when compared to that without FES [24], where a treatment duration beyond 12 weeks might have shown additional gains. In this preliminary case study in India we studied (1) user response to the latest version of the Odstock Dropped Foot Stimulator (ODFS, Odstock, UK) [18] called ODFS Pace as an orthotic intervention, (2) short-duration moderate-intensity FES-assisted gait training as a therapeutic intervention at the rehabilitation clinic. We investigated changes in the volitional gait from baseline on 7 chronic (> 6 months after stroke) stroke survivors following 6 sessions (3 times a week, for 2 weeks) of 30 minutes of FES-assisted treadmill walking at their comfortable walking speed.

## 2. Case Series

The single-channel ODFS Pace (Odstock, UK) provides heel-switch triggered functional electrical stimulation (FES) to assist dorsiflexion of the ankle during walking [18]. The ODFS was recommended in the year 2000 as an intervention for neurologically impaired gait in the Clinical Guidelines on Stroke by the Royal College of Physicians (UK) and over 2000 patients have been fitted with ODFS for foot drop in UK. Based on the guidelines by the National Institute of Clinical Excellence (NICE, UK) which recommended (in July 2008) dropped foot stimulators as an intervention in stroke (http://www.nice.org.uk/Guidelines/IPG278/), we applied for ethics approval and started the first clinical study on ODFS Pace in India at the Max Super Speciality Hospitals Saket, India.

Four male and three female hemiplegic stroke survivors (age: 31–76 years) volunteered for this study. The study was approved by the Institutional Review Board of the Max Super Speciality Hospitals, Saket, India (http://www.ctri.nic.in/Clinicaltrials/pmaindet2.php?trialid=3821). Informed consent was obtained from all the subjects before their participation. The inclusion and exclusion criteria for the study are listed in Table 1. The ODFS Pace electrically stimulated the common peroneal nerve or the neuromuscular junction of the tibialis anterior (TA) muscle via self-adhesive skin electrodes which were positioned on the lower leg [18]. The placement of the stimulating electrodes is critical for balanced dorsiflexion and a trained physiotherapist fitted the stimulator to the patients. The stimulation was timed to the walking cycle by use of a switch which was placed under the heel. The stimulation was timed to begin right after or after a small delay when the heel was lifted from the ground and ended right after or after a small delay following heel strike. The electrical stimulation activated the muscles related to ankle dorsiflexion and caused the foot to lift when the user lifted the leg to step forward [18]. By extending the stimulation beyond heel strike, the stimulation stabilized the ankle when the weight was loaded on the foot (i.e., loading phase).

After the baseline assessments to verify the suitability of the prospective subjects based on our inclusion/exclusion criteria (presented in Table 1), 7 subjects were recruited for the FES-assisted gait training every alternate day for 2 weeks (i.e., thrice a week) in which 30 minutes of treadmill-walking and simultaneous “naked eye” quick observational gait analysis (OGA) [27] to adjust stimulation parameters, were performed under the supervision of a trained physiotherapist. Since interrater agreement is much higher with a binary scale [28, 29] therefore a trained physiotherapist rated gait deviations at the Ankle, Knee, Hip, Pelvis, and Trunk as “present” or “absent.” The manual muscle test (MMT) on the affected ankle dorsiflexor (tibialis anterior) muscle and a measure of ankle spasticity on modified Ashworth scale (MAS) [30] were also performed before FES-assisted gait training. We investigated incremental changes (if any) in the volitional gait using pre- versus postintervention video-based OGA [27–29]. We also conducted patient survey to record their comments and subjective assessments based on Usability Rating Scale (URS) [31]. The subjective assessments was conducted on a 7-point scale with URS, of the perceived ease of use of ODFS Pace for heel switch triggered FES-assisted walking. The subject was asked to rate the ODFS Pace for FES-assisted walking as “difficult,” “moderate,” or “easy.” After selecting one of those three choices, the subject was asked to refine his/her selection as “very,” “moderately,” or “barely.” The case series is described below and the diagnostic scores are summarized in Table 2.

**Case 1**

A 31-year-old young, energetic, and moderately obese gentleman suffered stroke in July 2008 (3 years, 8 months after stroke) which resulted in left hemiplegia. The subject complained of putting on weight due to sedentary lifestyle since the instability in his affected ankle led to decreased mobility. He was recommended FES-assisted gait therapy to develop confidence for community ambulation, to improve cardiovascular endurance, and possible therapeutic benefits. His preintervention video-based OGA of the affected side showed lack of dorsiflexion at heel strike resulting in foot slap, and mostly inverted foot during swing phase. Also, he had inadequate knee flexion during swing phase, and hip hiking with inadequate hip flexion during terminal swing phase. The pelvis and trunk however showed no significant deviations from normal. He underwent our two weeks of FES-assisted gait training as described earlier. His postintervention video-based OGA of the affected side during volitional walking showed improved toe off during terminal stance phase, improvement in knee flexion during swing phase, but no significant changes at the hip from preintervention. The subject graded “easy” on URS and reported less-perceived effort during FES-assisted walking.

**Case 2**

A 58-year-old, moderately obese lady with coronary artery disease leading to percutaneous transluminal coronary angioplasty (PTCA), hypertension, diabetes mellitus, and acute renal failure due to diabetic nephropathy, suffered stroke in April 2009 (3 years after stroke) resulting in right hemiplegia. The subject complained of inability to walk without support and was dependent on her caregiver for activities of daily living (ADL). She was recommended FES-assisted gait therapy to develop confidence during walking and to reduce dependence on her caregiver for ADL. Her preintervention video-based OGA of the affected side during volitional walking showed lack of dorsiflexion at heel strike resulting in foot slap, and inverted foot during swing phase. She also had inadequate knee flexion during swing phase, inadequate hip flexion during terminal swing, and significant hip hiking during swing phase. She however had no significant deviation from normal at the pelvis and trunk. She underwent our two weeks of FES-assisted gait training as described earlier. Her postintervention video-based OGA of the affected side during volitional walking showed no significant change from preintervention. The subject however perceived “the leg is lighter” and “lesser twist in the foot” during FES-assisted walking. She graded “easy” on URS.

**Case 3**

A 72-year-old outgoing lady with hypertension suffered stroke in 2006 (6 years after stroke), which resulted in left hemiparesis. The subject recently complained of falls due to ankle instability. Due to fear of falls she had low confidence in activities outside her home for ADL. She was recommended FES-assisted gait therapy to develop confidence during walking and for therapeutic benefit. Her preintervention video-based OGA of the affected side during volitional walking showed lack of heel strike (i.e., foot slap) and inadequate toe off, lack of dorsiflexion during midswing to terminal swing, and inadequate ground clearance. There was also inadequate knee extension during midstance, inadequate knee flexion during swing phase, inadequate contralateral hip extension and improper hip flexion during swing phase, and circumduction during midswing. There was a lack of pelvic rotation during midswing to terminal swing, and a forward lean of the trunk during stance phase. She underwent our two weeks of FES-assisted gait training as described earlier. Her postintervention video-based OGA of the affected side during volitional walking showed improved ground clearance of the foot, improved knee flexion during swing phase, and improved hip flexion during the swing phase. She commented during FES-assisted walking that “I am able to lift my foot,” “I can walk more confidently,” “My walking speed has increased.” She graded “moderately difficult” on URS.

**Case 4**

A 63-year-old gentleman with hypertension and diabetes mellitus type II suffered stroke in August 2009 (2 years, 7 months after stroke) which resulted in left hemiplegia. He complained of labored walking. He was undergoing regular rehabilitation for last 15 months but showed no improvement. He was recommended FES-assisted gait therapy for possible therapeutic benefits. His preintervention video-based OGA of the affected side during volitional walking showed ground contact with forefoot, flat foot during the loading phase, and lack of dorsiflexion during swing phase. The knee was hyperextended during midstance and the subject suffered from reduced knee flexion during swing phase. He also suffered from hip hiking during swing phase, external rotation of the affected limb during toe off, and circumduction from preswing to terminal swing phase. The affected left pelvis hiked towards right from preswing to terminal swing, and there was no forward rotation of the pelvis during initial swing to terminal swing phase. There was also significant backward lean in the trunk from stance to preswing phase. He underwent our two weeks of FES-assisted gait training as described earlier. His postintervention video-based OGA of the affected side during volitional walking showed no significant change from preintervention. The subject liked FES-assisted treadmill walking where the foot assisted by FES could clear the ground more easily during the swing phase. However the rotation of the leg was still present during FES-assisted treadmill walking which demonstrated a weakness at the hip which couldnot be alleviated with the single-channel ODFS Pace. The subject graded “easy” on URS.

**Case 5**

A 73-year-old gentleman with coronary artery bypass graft (CABG) had a stroke in November 2010 (2 years, 3 months after stroke) which resulted in right hemiplegia. His primary concern was propensity for fall on uneven surfaces due to reduced ground clearance with foot drop. He was recommended FES-assisted gait therapy to develop confidence during walking and possible therapeutic benefits. His preintervention video-based OGA of the affected side during volitional walking showed ground contact with foot flat continuing into the loading phase, inadequate toe off, and inadequate dorsiflexion during swing phase. There was also inadequate knee flexion during terminal stance, and inadequate knee extension at the heel strike. There was inadequate hip extension from midstance to preswing, and slight circumduction from preswing to terminal swing phase. There were however no significant deviations at the pelvis or trunk. He underwent our two weeks of FES-assisted gait training as described earlier. His postintervention video-based OGA of the affected side during volitional walking showed improved toe off, improved knee flexion in swing phase, and improved hip extension. The subject remarked—“easy to lift foot with FES”—during his gait training. The subject graded “easy” on URS.

**Case 6**

A 76-year-old female with CABG had a stroke in November 2009 (2 years, 3 months after stroke) resulting in left hemiplegia. Her primary concern was weakness in the affected ankle. She was recommended FES-assisted gait therapy to develop confidence during walking and possible therapeutic benefits. Her preintervention video-based OGA of the affected side during volitional walking showed ground contact with foot flat continuing in the loading phase, inadequate knee flexion during swing phase, inadequate hip flexion during midstance, slight circumduction from preswing to terminal swing, inadequate pelvic rotation, and hip hiking from preswing to terminal swing. The trunk however showed no significant deviation from normal. She underwent our two weeks of FES-assisted gait training as described earlier. Her postintervention video-based OGA of the affected side during volitional walking showed no significant change when compared to preintervention. The subject however reported less effort during walking with FES-assistance and graded “easy” on URS.

**Case 7**

A 65-year-old physically active gentleman suffered stroke in October 2009 (2 years, 5 months after stroke) resulting in right hemiplegia. His primary concern was labored walking and foot drop which was corrected for ADL with an AFO, resulting in muscle atrophy at the ankle. He was recommended FES-assisted gait therapy to reduce muscle atrophy and possible therapeutic benefits. His preintervention video-based OGA of the affected side during volitional walking showed ground contact with forefoot or foot flat continuing in the loading phase, and lack of dorsiflexion. There was also inadequate knee flexion during swing phase, inadequate hip extension from midstance to terminal swing phase, and slight circumduction from preswing to terminal swing phase. There was a lack of pelvic rotation, significant hip hiking from initial swing to mid-swing, and lack of lateral horizontal shift of the trunk during stance phase. He underwent our two weeks of FES-assisted gait training as described earlier. His postintervention video-based OGA of the affected side during volitional walking however showed no significant change from preintervention. The subject liked FES-assistance to lift the foot during treadmill walking. He graded “easy” on URS.

## 3. Discussion

We found that our short-duration, moderately intensive FES-assisted gait therapy improved volitional gait in 3 out of 7 stroke survivors suffering from foot drop. Even though prior work [17] showed a treatment duration beyond 12 weeks necessary for reaching a plateau for therapeutic benefits, we kept the duration of the FES-assisted gait therapy short (only 2 weeks) to monitor only incremental change and didnot want the therapeutic benefits to plateau before a planned randomized controlled crossover study on electromyogram-triggered FES-assisted gait training [32]. Even in absence of improvement in volitional walking following FES-assisted gait therapy, there were no adverse effects and the subjects found heel-switch triggered FES-assisted gait with ODFS Pace mostly “easy” (6 out of 7 subjects) on URS. We postulate that the “carry over” effect that we intended to observe following short-duration, moderately intensive FES-assisted gait therapy is patient specific and we need to perform a case-by-case study with a larger cohort in future. 

In published reports, the functional integrity of the corticospinal tracts (CSTs) has been associated with an individuals capacity for further functional improvement following stroke [33–35]. Moreover, stroke presents with heterogeneous patient-specific impairments in motor, sensory, tone, visual, perceptual, cognition, aphasia, apraxia, coordination, and equilibrium. Therefore the functional limitations following stroke are varied, including gait dysfunction, fall risk, limited activities of daily living, difficulties in swallowing, reduced upper extremity function, altered communication, besides others. Based on the residual function of a stroke survivor, the number of muscles that need FES assistance, intensity, frequency, and duration of the FES-assisted gait training need to be decided. The ability of a stroke survivor to undergo FES-assisted gait therapy also depends on their cardiovascular and neuromuscular capacity besides psychological factors such as motivation. We found that one channel of FES that is available with ODFS Pace was not enough to provide functional gait to some of our subjects (e.g., Cases 3, 4, 6, and 7), who needed more channels due to significant deviations in pelvis and trunk gait trajectories from normal as found from preintervention video-based OGA. Therefore in those cases, the therapeutic benefits may have been limited by the lack of gait training with multijoint coordination assisted with multichannel FES [25]. 

It has been shown that only 10% of stroke survivors recover limb strength and mobility rapidly enough to prevent contractures [36]. Also, 20% of stroke survivors suffer muscle atrophy which underlie worsening metabolic fitness in the chronic phase of stroke including gross muscular atrophy, altered muscle molecular phenotype, increased intramuscular area fat, elevated tissue inflammatory markers, and diminished peripheral blood flow dynamics [37]. Moreover, bone resorption begins after 30 hours of immobility which results in 17% bone loss in the paretic arm and up to 12% in the paretic leg in the chronic stages of stroke [38]. Therefore hip fracture, which is the most frequent fracture following stroke, can have incidences 2–4 times higher in stroke patients compared with the reference population [39]. FES-assisted gait training may alleviate these debilitating conditions where increased intensity and frequency of rehabilitation may help [40]. In fact recent studies in India on therapeutic benefits of FES-assisted gait training in conjunction with conventional physiotherapy have shown the additive effects of FES on reducing spasticity, improving dorsiflexor strength, improving walking ability, and improving metabolic fitness [41–43].

However lack of accessible rehabilitation facilities and high cost of rehabilitation at the well-equipped clinics are current challenges in India which usually lead to high-dropout of individuals from rehabilitation therapy following stroke. Any deconditioned chronic stroke survivor will need to recondition their cardiovascular endurance, metabolic fitness, muscle conditions, and bone strength with a gradual increase in the intensity (number of hours per day) and frequency (number of days per week) of FES-assisted gait therapy, providing a higher level as they improve their function. Also, the FES-assisted gait training should be started as soon as the patient becomes clinically stable since early intervention has shown better functional outcomes, survival rates, and reduced length of required therapy [44]. Therefore we are currently investigating a home-based rehabilitation model where stroke survivors can use a low-cost FES device (such as ODFS Pace) as an orthotic intervention for daily use during walking at home and subsequently for community ambulation after they achieve proficiency. Following a short stay in rehabilitation clinic, home-based FES-assisted treadmill-gait therapy may also be more economical and feasible, with a consulting physiotherapist in case of more affected stroke survivors. However these home-based models need to be validated with stroke survivors in India where well-equipped rehabilitation clinics are expensive, far, and few.

 In closing, we would summarize our research hypothesis for stroke neurorehabilitation following Ward and Cohen [15], who have presented lessons from animal models where manipulation of environmental, behavioral, and pharmacologic contexts influenced cerebral reorganization and consequently the process of recovery of function following stroke. We propose that early task-specific FES-assisted gait therapy may drive functionally relevant neuroplastic changes in the brain. However such FES-assisted gait therapy needs to be tailored to individual health condition as identified based on WHO International Classification of Functioning (ICF). The WHO ICF model recommends intervention at multiple levels (e.g., impairment, activity, participation) where environment and personal factors can play an important role. We also need to measure at each level for example, individuals capacity for functional recovery measured with imaging techniques [26–28], metabolic fitness, and cardiovascular health measured with physiological cost index [31], and functional gait analysis to select the paretic muscles to be assisted with FES. With the WHO ICF model, we may be able to understand the response to the FES-assisted gait therapy at each level, and also understand the relationships between different levels for future planning.

## Supplementary Material

The video is an example of heel-switch triggered functional electrical stimulation assisted overground ambulation with single-channel ODFS Pace (Odstock, UK)

## Figures and Tables

**Table 1:** Inclusion and exclusion criteria for the stroke study.

Inclusion criteria	Exclusion criteria
Age 21 to 80 years	Brainstem stroke
>6 months from a first clinical nonhemorrhagic or hemorrhagic stroke	Epilepsy
Medically stable	Severely impaired cognition and communication
Unilateral lower extremity hemiparesis	History of peroneal nerve injury
Able to ambulate 16 feet (5 meters) continuously with minimal assistance or less, without the use of an Ankle Foot Orthosis (AFO)	History of Parkinson's, spinal cord injury, traumatic brain injury, multiple sclerosis, and uncontrolled seizure disorder
AFO is clinically indicated (foot drop during ambulation)	Uncompensated hemineglect (extinguishing to double simultaneous stimulation)
Electrical stimulation of the paretic ankle dorsiflexors produces ankle dorsiflexion to neutral without pain	Edema of the paretic lower extremity
Full-voluntary dorsiflexion of the contralateral ankle	Absent sensation of lower leg and foot
Skin intact on hemiparetic lower extremity	History of cardiac arrhythmias with hemodynamic instability
	Cardiac pacemaker or other implanted electronic system
	Botulinum toxin injections to any lower-extremity muscle in the last 3 months
	Evidence of deep venous thrombosis or thromboembolism

**Table 2:** Summary of the case series (M: male, F: female, MCA: middle cerebral artery, PTCA: percutaneous transluminal coronary angioplasty, CABG: coronary artery bypass graft, TA: tibialis anterior muscle, MMT: manual muscle test, MAS: modified Ashworth scale, URS: usability rating scale, OGA: observational gait analysis, MRI: magnetic resonance imaging).

Case	Age/gender	MRI diagnosis	Comorbidities	Year of stroke	TA MMT	Ankle MAS	URS	Pre versus postintervention OGA
1	31/M	Right MCA stroke: infarct in right basal ganglia, fronto-temporal, and perisylvian grey and white matter.	None	2008	1+	3	Easy	Improvement

2	58/F	Left MCA stroke: acute non-hemorrhagic infarct in left basal ganglia and paraventricular white matter/corona radiate with lacunar infarct in left high frontal pre-central cortex.	Diabetes, hypertension, post-PTCA	2009	2−	2	Easy	No change

3	72/F	Right basal ganglia infarct: gliotic area and old hemorrhagic remnant in right basal ganglia and thalamus with chronic ischemic changes in the brain.	Hypertension	2006	3−	2	Moderately difficult	Improvement

4	63/M	Left MCA stroke including basal ganglia: infarct in the territory supplied by left Middle Cerebral Artery (MCA) including basal ganglia.	Diabetes mellitus type II, hypertension	2009	2	2	Easy	No change

5	73/M	Left MCA stroke.	Post-CABG	2010	3−	1+	Easy	Improvement

6	76/F	Right MCA stroke, including basal ganglia.	Post-CABG	2009	3−	1+	Easy	No change

7	65/F	Left MCA stroke, including basal ganglia and subcortical white matter.	None	2009	1	3	Easy	No change
